# Exploring the Use of Interleaved Stimuli to Measure Cochlear-Implant Excitation Patterns

**DOI:** 10.1007/s10162-024-00937-2

**Published:** 2024-03-08

**Authors:** François Guérit, John C. Middlebrooks, Robin Gransier, Matthew L. Richardson, Jan Wouters, Robert P. Carlyon

**Affiliations:** 1 Cambridge Hearing Group, MRC Cognition & Brain Sciences Unit, University of Cambridge, Cambridge, England; 2 Department of Otolaryngology, University of California at Irvine, Irvine, CA USA; 3 Department of Neurobiology and Behavior, University of California at Irvine, Irvine, CA USA; 4 Department of Biomedical Engineering, University of California at Irvine, Irvine, CA USA; 5 Department of Neurosciences, ExpORL KU Leuven, Leuven, Belgium; 6 Leuven Brain Institute KU Leuven, Leuven, Belgium

**Keywords:** Cochlear implants, Excitation patterns, Interleaved masking, Monopolar, Tripolar

## Abstract

**Purpose:**

Attempts to use current-focussing strategies with cochlear implants (CI) to reduce neural spread-of-excitation have met with only mixed success in human studies, in contrast to promising results in animal studies. Although this discrepancy could stem from between-species anatomical and aetiological differences, the masking experiments used in human studies may be insufficiently sensitive to differences in excitation-pattern width.

**Methods:**

We used an interleaved-masking method to measure psychophysical excitation patterns in seven participants with four masker stimulation configurations: monopolar (MP), partial tripolar (pTP), a wider partial tripolar (pTP + 2), and, importantly, a condition (RP + 2) designed to produce a broader excitation pattern than MP. The probe was always in partial-tripolar configuration.

**Results:**

We found a significant effect of stimulation configuration on both the amount of on-site masking (mask and probe on same electrode; an indirect indicator of sharpness) and the difference between off-site and on-site masking. Differences were driven solely by RP + 2 producing a broader excitation pattern than the other configurations, whereas monopolar and the two current-focussing configurations did not statistically differ from each other.

**Conclusion:**

A method that is sensitive enough to reveal a modest broadening in RP + 2 showed no evidence for sharpening with focussed stimulation. We also showed that although voltage recordings from the implant accurately predicted a broadening of the psychophysical excitation patterns with RP + 2, they wrongly predicted a strong sharpening with pTP + 2. We additionally argue, based on our recent research, that the interleaved-masking method can usefully be applied to non-human species and objective measures of CI excitation patterns.

## Introduction

**Fig. 1:**
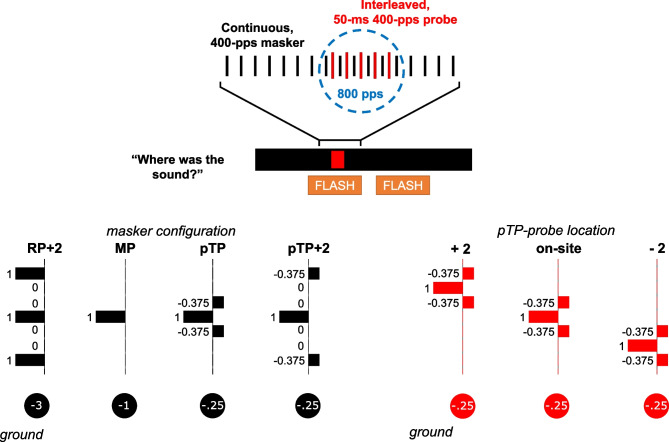
*Top*: interleaved psychophysical masking paradigm used throughout the study. The probe was always a 50-ms, 400-pps pulse train (i.e. 20 pulses, although only 5 pulses are shown in the schematic) presented in pTP configuration. In the masked conditions, the masker (continuous, at 400 pps) and probe were interleaved so that it effectively was an isochronous 800-pps pulse train for 50 ms. Visual flashing lasted 200 ms before and after the probe. *Bottom*: schematics of the various stimulation configurations used for the continuous masker. pTP stands for partial tripolar (75% of the current returned to the two-nearest electrode, the rest at the external case electrode); pTP + 2 for partial tripolar with 75% of the current returned to electrodes located three electrodes away from the centre one; MP for monopolar (100% of the current returned through the external case electrode), and RP + 2 for ralopirt (tripolar written backwards), a ‘broad monopolar’ configuration where three electrodes are stimulated in phase. Circles at the bottom show how much current is returned through the external case electrode of the implant

Arguably, one of the greatest obstacles to speech perception by cochlear implant (CI) listeners, especially in noisy situations, is the broad spread of neural excitation produced by stimulating CI electrodes [1, 2]. Most contemporary CIs use the monopolar (MP) configuration of stimulation, whereby the current injected by each electrode is returned by an electrode located outside of the cochlea. The resulting broad current spread and broad neural excitation patterns along the length of the cochlea have prompted the investigation of potentially more-focussed forms of stimulation, including the tripolar (TP) and partial tripolar (pTP) configurations (Fig. 1). These stimulation configurations have revealed substantial improvements in spatial (tonotopic) selectivity in recordings from anaesthetised animals [3, 4], but only mixed and at best modest improvements in psychophysical and speech-perception experiments with humans (see [5], for a review).

As a first step towards resolving the discrepancy between the human and animal results, we developed a non-invasive electrophysiological method for measuring masked excitation patterns in NH humans and cats, which was validated against psychophysical measures in the two species [6]. That paradigm involved scalp-based recordings of the cortical onset response to tones of different frequencies presented against a continuous noise masker. We chose simultaneous rather than forward masking so as to avoid contamination of the response to probe onsets by responses to masker offsets. Here, we use a CI-analogue of simultaneous masking to compare psychophysical masked excitation patterns for maskers varying in configuration, namely, the interleaved-masking paradigm illustrated in Fig. 1. Results from previous psychophysical studies using interleaved masking highlight the importance of minimising charge interactions between masker and probe pulses, which have been observed for pulse separations shorter than 0.5 ms [7, 8], and so, we ensured that masker and probe pulses were separated by 1.25 ms (centre to centre) and additionally included a check for such interactions. Other advantages of using interleaved masking are that it reflects a situation encountered in everyday (clinical) CI use and that it may be less prone to the confusion effects that can arise in forward masking experiments when a brief probe follows a longer masker after a short interval. These confusion effects can occur when the masker and probe have similar temporal properties and excitation patterns, causing the probe to be perceived primarily as a continuation of the masker [7, 9]. Finally, we were careful to adjust the levels of all maskers to produce approximately equal amounts of masking for a probe presented to the same electrode as the masker. Equating this ‘on site’ masking permits a straightforward comparison of masked excitation patterns for different maskers. When on-site masking is not equated it is possible to scale the different excitation patterns to align at their peaks, but the choice of scale (linear, logarithmic, or other) can then affect which pattern is judged to be sharper [7].

The present study investigated two further possible reasons for the variable and modest success of attempts to improve tonotopic selectivity in CI users. The first reason is that the focussed-stimulation stimuli tested so far may not be optimal for minimising the spread of excitation. The vast majority of investigations have used either bipolar (BP), TP, or pTP stimuli; pTP typically is used rather than full TP because of the impractically high currents that are needed to obtain thresholds and comfortable levels with full TP. BP stimuli can produce narrower current spread than MP but, because each electrode is stimulated by the same current amplitude, the resulting excitation patterns can be bimodal [10–12]. This problem is reduced with TP and pTP, but ‘side lobes’ of excitation can appear, at least in computational models [13, 14], and the practice of returning current from the adjacent electrodes (Fig. 1) may not be ideal for minimising spread of excitation. For example, Luo and Wu [15] have reported that returning current from the adjacent-but-one electrodes can reduce both current spread and the width of forward-masked excitation patterns compared to the standard pTP method. Our intra-cochlear voltage recordings, described below, indicated that leaving two unused electrodes between the central and flanking electrodes can produce reduced current spread compared to ‘standard’ pTP. We therefore compare masked excitation patterns for this pTP + 2 stimulus to those produced by MP maskers and by ‘standard’ pTP maskers. Second, a failure to observe a difference between two maskers might be due either to those maskers producing similar excitation patterns or alternatively to a lack of sensitivity or power in our experimental design. We therefore included a condition (‘RP + 2’, Fig. 1) that has a broader current spread than the other maskers along the electrode array. We reasoned that, if we could measure a broadened masked excitation pattern for this stimulus, then it would demonstrate the ability of our paradigm to detect excitation-pattern differences. We found that this was indeed the case and confirmed this finding using an alternative single-point measure of excitation pattern width according to which, for two maskers of equal loudness, on-site masking should be greater for the masker producing the narrower excitation pattern [10].

## Methods

### General Methods

Experimental procedures were approved by the National Research Ethics Committee for the East of England (ref. number 00/327), and written informed consent was collected prior to any testing. All experiments were performed with participants implanted with a CI manufactured by the Advanced Bionics company. Information on each participant and on the centre electrode of the maskers tested is shown in Table 1. CI electrodes were stimulated by bypassing the clinical processor and connecting the radio-frequency coil to a Platinum Series Processor provided by Advanced Bionics (Valencia, USA), which in turn was connected to a laptop computer. Stimuli were generated using programs written in Matlab and that called routines from the Bionic Ear Data Collection System provided by Advanced Bionics.

**Table 1:** Participant details. 1j is a straight electrode array; ms (‘mid-scala’) is a pre-curved electrode array

**ID**	**Age**	**Ear**	**Electrode**	**Masker EL**	**Pilot exp**	**Main exp**
AB1	74	Left	1j	8	X	X
AB2	60	Left	1j	4	X	X
AB3	73	Left	1j	4	X	
AB5	76	Left	1j	4		X
AB6	70	Right	1j	4	X	
AB13	87	Right	1j	4		X
AB23	60	Right	ms	4		X
AB24	50	Left	ms	4		X
AB26	58	Left	ms	4		X

As shown in Fig. 1, listeners were required to detect a 400-pps 50-ms pulse train (the probe) interleaved with various masker types. The masker centre electrode was fixed throughout all experiments, and the probe centre electrode was either the same as the masker centre electrode or shifted by 2 electrodes more basally/apically. This probe was presented in partial tripolar (pTP) configuration, with current injected via a central electrode and with 75% of that current returned through two adjacent flanking electrodes; the remaining 25% was returned through the case of the implant (Fig. 1). In each 2-interval 2-alternative forced-choice trial, the listener indicated which of two intervals, marked by flashing virtual buttons on a computer screen, contained the probe. When a masker was present, it had a pulse rate of 400 pps, and the masker and probe were interleaved so that the probe pulses fell exactly mid-way between adjacent masker pulses leading to a centre-to-centre inter-pulse interval for the combined stimulus of 1.25 ms﻿ (Fig. 1). This was chosen such that the inter-pulse interval was long enough to minimise charge interactions, but short enough such that the individual and combined pulse rates fell above the “upper limit” of temporal pitch  (~300-400 pps). The strength of any pitch change introduced by the probe would presumably have been greatest when the probe and masker pulses were equally loud, and we wished to avoid the nature of the cue changing during an adaptive track. The masker had a duration of 2.4 s: 500 ms of masker-only pre-stimulation, 450 ms for the first interval (200 ms masker-only, 50 ms masker + probe, 200 ms masker-only), 500 ms of inter-interval stimulation, 450 ms for the second interval, and 500 ms of masker-only to end with. All stimuli consisted of trains of symmetric biphasic pulses having phase durations of 97 µs and with zero inter-phase gap. Probe thresholds were estimated using a ‘1-up-2-down’ adaptive procedure in which probe level was increased after every incorrect answer and decreased after every two consecutive correct answers, thereby converging on the 70%-correct point of the underlying psychometric function [16]. The change from decreasing to increasing probe level or vice versa defined a turn point. The size of the level steps were 1 dB for the first 2 turn points and 0.5 dB thereafter. The procedure ended after the 8th turn point, and the probe levels from the last 6 turn points were averaged to obtain threshold for that run. Thresholds for each listener and condition were calculated from the average of three runs, or from four runs if thresholds for any two of the first three runs differed by more than 2 dB.

Statistical analyses were performed using IBM SPPS version 27. Mauchly’s test of sphericity was used to check for equality of variance, where necessary *p* values and effect sizes were adjusted using the Huynh–Feldt correction and are reported along with the original degrees of freedom. Effect sizes (*η*_p_^2^) are reported for all significant effects and interactions. For analysis of post hoc comparisons, we always used the Bonferonni correction for multiple comparisons and report this by multiplying the ‘raw’ *p* value by the number of comparisons made.

### Preliminary Experiment: Charge Interactions

Four listeners took part in a preliminary experiment to check for facilitation effects between the masker and the probe. Facilitation effects are stronger when the second phase of one biphasic pulse has the same polarity as the first phase of a following pulse [7, 17–19]. In our main experiment, the masker second phase and the probe first phase are of opposite polarity (both consisted of cathodic-leading biphasic pulses), but we verify here that flipping the polarity of either the probe or the masker does not change thresholds, as this would be a sign that facilitation could be occurring at the inter-pulse interval we use (1.25 ms). We first measured the Most Comfortable Loudness level (MCL) for the MP and pTP maskers. To do this, the masker durations were reduced to 500 ms. The experimenter played the stimuli, and the listeners pointed on an 11-point scale ranging from ‘inaudible’ to ‘too loud’, with the MCL corresponding to point 7. The experimenter always started with very soft stimuli and increased the level until point 7 was reached, making sure to go down and up again in level to confirm the listener’s judgement before deciding on the final MCL. The MCL was obtained in this way both for pulse trains with anodic-leading and cathodic-leading polarity. A unique level, slightly below the MCLs of each polarity, was picked for the masked detection thresholds; note that for each masker type (MP or pTP), the level was the same for both polarities. The MCLs for the 50-ms pTP probe were similarly obtained for both polarities. Next, masked on-site thresholds for the probe were obtained for all four combinations of masker and probe polarity, as described in the ‘General Methods’ section and using the full 2.4-s masker duration.

### Main Experiment

#### Masked Excitation Patterns

The main experiment measured probe thresholds in quiet and in the presence of four different maskers, each of which is illustrated in Fig. 1. The MP and pTP maskers were the same as described above. The pTP + 2 masker was similar to the pTP masker except that the flanking electrodes were separated from the central electrode by two unused electrodes. The electrodes used in the RP + 2 stimulus were the same as for pTP + 2, but stimulated in the same (rather than opposite) polarity and with equal amplitude applied to all three electrodes (The abbreviation RP stands for ‘ralopirt’, which is the word ‘tripolar’ spelled backwards). The electrode location of the RP + 2 complex is given by the centre of the three source electrodes. All maskers as well as the probe consisted of cathodic-leading biphasic pulses.

The first part of the experiment measured on-site masking for seven listeners in two conditions. In the first condition, the maskers were set to have equal loudness. It started with a loudness-scaling procedure identical to that used in the preliminary experiment and using shortened, 500-ms versions of each masker. The maskers were then loudness-balanced to each other using a procedure based on the one described by Landsberger and McKay [20]. Initially, the level of the MP stimulus was fixed to its MCL and presented first (the reference) in a pair with another masker type (the test). The listener was instructed to report whether the test sound was quieter, at the same loudness, or louder than the reference. The experimenter then increased and/or decreased the level of the test stimulus until the participant was confident that both sounds were at equal loudness, making sure to first bracket the level of the test stimulus in order to yield softer and louder percept than the reference stimulus. The whole procedure/run was repeated four times, each with a slightly different initial level of the test stimulus, and each time swapping the stimulus type (i.e. MP stimulus became the test if it was the reference in the previous run, and vice versa). The matched loudness of the test stimulus was then taken as the level of MP stimulus plus the mean level difference obtained from all four matches. The second condition measured the changes in masker level needed to produce approximately equal on-site masking for all maskers. To do this, any masked thresholds for the pTP, pTP + 2, and RP + 2 maskers that differed by more than 1 dB from the MP masker in the first condition were re-measured (i.e. a full set of 3–4 threshold measurements) with the masker level adjusted by a small amount (typically 0.25 or 0.5 dB). In some cases, the level of the masker had to be adjusted several times for the masked threshold to differ by less than 1 dB from the MP-masker condition.

In previous studies and in some preliminary measures, we used a different method for equating on-site masking, in which the levels of the different on-site maskers were varied throughout using an adaptive procedure so as to just mask the probe. We found that although this method sometimes worked well with the stimuli used here, it also sometimes produced quite variable results and with erratic adaptive procedure tracks; furthermore, the masked thresholds subsequently measured by adaptively varying the probe level were not, as intended, always the same for all maskers. Procedural difficulties in equating on-site masking using a masker-varying method have also been reported in an earlier study [10]. We reasoned this may have been due to listeners attending primarily to the masker, which varied from trial to trial, rather than to the probe which was fixed in level throughout each run. The method that we finally adopted, although relying on the experimenter’s best estimates of what level to try next, produced a pragmatic solution as reflected in reliable thresholds and monotonic underlying psychometric functions (not shown here).

The second part of the experiment measured masked excitation patterns using the equally effective (on-site) masker levels obtained in the first part. To do this, masked thresholds were obtained for probe locations that were 2 electrodes apical and 2 electrodes basal to the masker electrode (or to the central electrode of the RP + 2 configuration) and by combining these with the on-site masked thresholds from the first part. In this configuration, the active electrode of the probe was one electrode displaced from the flanking electrodes of the pTP + 2 and RP + 2 configurations (Fig. 1). Probe thresholds for each electrode in the absence of the masker were also obtained so that masked thresholds could be expressed as the amount of masking in dB relative to the unmasked thresholds.

#### Cochlear Voltage Recordings

For all participants and masker types, we measured the resulting stimulation voltage along the whole length of the electrode array at the stimulation levels of the main experiment. To do so, we first obtained Stimulation-Current-Induced Non-Stimulating Electrode Voltage recordings (‘SCINSEVs’ [21]) using the Volta software provided by Advanced Bionics, who use the term ‘Electric Field Interactions’ for this type of measurement: each electrode was stimulated in monopolar configuration, with the resulting voltage recorded at all the electrodes. This yielded a 16-by-16 matrix with all possible combinations of stimulating and recording electrode, which was then normalised by the input current to give values in ohms. The diagonal of this matrix **Z** (stimulating and recording on the same electrode) does not reflect the true voltage at the electrode array, because of the contact impedance contributing to the values being measured [22, 23]. The underlying voltage can however be estimated with linear extrapolation [23] or a ladder-network of resistors [22]. We used the linear extrapolation method described by van den Honert and Kelsall [23] for simplicity. Once the diagonal values were estimated, and assuming linearity, we could estimate the voltage at each electrode for any stimulation modality and level by matrix multiplication: **V** = *Z* * **I**, where **V** is the resulting voltage, *Z* the 16-by-16 matrix, and **I** the injected current at the different electrodes. The linearity assumption is valid as long as measures are within compliance limits of the device [24, 25]. As common practice in our laboratory and for safety reasons, we always measure limits of compliance prior to and after any testing. As a check, we also measured the voltages for each masker stimulation configuration at the non-stimulating electrodes using the Advanced Bionics ‘BEDCS’ software; these voltages were very similar to the voltages estimated from the matrix multiplication, apart from an overall difference in gain due to measuring the voltages at a different time point in the waveform.

## Results

### Preliminary Experiment: Charge Interactions

**Fig. 2:**
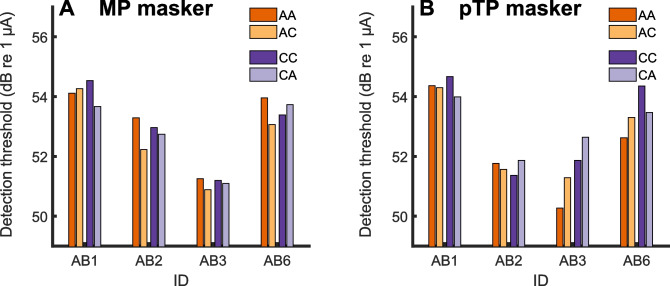
Results of the preliminary experiment checking for charge interactions. With interleaved masking, there is a risk that at short inter-pulse intervals between the probe and masker, the masker would facilitate perception of the probe. This can be checked by flipping the relative polarity of the masker and probe pulses: such facilitation will be increased when the second phase of the masker and the first phase of the probe are of same polarity. **A** Polarity check with the monopolar masker. Dark orange and dark purple show detection thresholds for the masker and probe having anodic (AA) or cathodic (CC) first-phase polarity, respectively. Light orange and light purple show the results where the masker and probe have opposite first-phase polarity (AC, masker anodic-first, CA masker cathodic-first) in which case one would expect lower thresholds if the masker facilitates the probe. **B** Same results with a partial-tripolar masker (probe was partial-tripolar in both cases)

The masker levels used in the preliminary experiment are shown in Table 2, and were, not surprisingly, higher for the pTP than for the MP masker (*t*(3) = 8.0, *p* = 0.004). The masked thresholds for each combination of masker and probe polarity are shown for the MP and pTP maskers in Fig. 2A, B, respectively. An effect of charge interaction would be reflected by thresholds for the opposite-leading-polarity conditions (AC and CA; fainter-coloured bars) being lower than for the same-leading-polarity conditions (darker bars). This is because, for the opposite-leading-polarity stimuli, the second phase of one pulse would have the same polarity as the first phase of the next pulse, and so adding a probe to a masker in this configuration would increase loudness [26]. To test this prediction, we performed a three-way repeated-measures ANOVA with masker and probe polarity and masker configuration as factors. No significant effects or interactions were observed, including the two-way masker X probe polarity interaction (*F*(1,3) = 0.58, *p* = 0.50) and the three-way interaction between masker polarity, probe polarity, and masker configuration interaction (*F*(1,3) = 3.80, *p* = 0.15). We note, however, that these analyses were based on data from only four participants of this preliminary experiment; hence, although no evidence for interactions can be discerned from Fig. 2, we cannot rule out that possibility that there was no charge interaction at all.

**Table 2:** Masker levels used in the pilot experiment (polarity check)

**ID**	**MP masker level (dB re 1 µA)**	**pTP masker level (dB re 1 µA)**	**Level offset (pTP-MP)**
AB1	44.6	56.3	11.7
AB2	43.8	55.2	11.4
AB3	46	55.3	9.3
AB6	45.1	56.4	11.3
		**Mean**	**10.9**

### Main Experiment

#### Masked Excitation Patterns and On-Site Masking

The left-hand columns of Table 3 show the levels of the equally loud maskers obtained in the first part of the experiment, and the right-hand columns show the levels adjusted so as to produce approximately equal on-site masking. Nine out of the 21 combinations of masker type (pTP, pTP + 2, and RP + 2) met our criterion for requiring adjustment to produce approximately equal on-site masking as the MP masker, and these are indicated in bold italics in the table.

**Table 3:** Masker levels used in the main part of the experiment for each participant and averaged across participants, in dB re 1 µA. Data in the left-hand part are for equally loud maskers, whereas those on the right are for masker levels adjusted to produce approximately equal on-site masking, and with instances where this adjustment was necessary shown in bold italics (with the adjustment value shown in parentheses)

	**Equal loudness**				**Equal on-site masking**			
	**M**	**pTP**	**pTP + 2**	**RP + 2**	**M**	**pTP**	**pTP + 2**	**RP2**
AB1	45.1	57.0	52.7	36.4	45.1	57.0	52.7	36.4
AB2	45.5	57.0	52.9	36.7	45.5	57.0	52.9	***36.9 (*+ *0.2)***
AB5	44.1	55.7	52.8	35.1	44.1	55.7	***53.1 (*+ *0.3)***	***35.6 (*+ *0.5)***
AB13	45.6	57.7	54.7	36.7	45.6	57.7	54.7	36.7
AB23	44.6	53.3	48.9	36.9	44.6	***52.3 (− 1.0)***	48.9	***37.8 (*+ *0.9)***
AB24	47.6	55.5	51.3	39.9	47.6	***55.2 (− 0.3)***	***50.8 (− 0.5)***	***42.0 (*+ *2.1)***
AB26	44.5	55.6	53.1	35.8	44.5	***56.1 (*+ *0.5)***	53.1	35.8
**Mean**	**45.3**	**55.9**	**52.3**	**36.8**	**45.3**	**55.8**	**52.3**	**37.3**
**Std. dev**	*1.2*	*1.5*	*1.8*	*1.5*	*1.2*	*1.8*	*1.9*	*2.2*

**Fig. 3:**
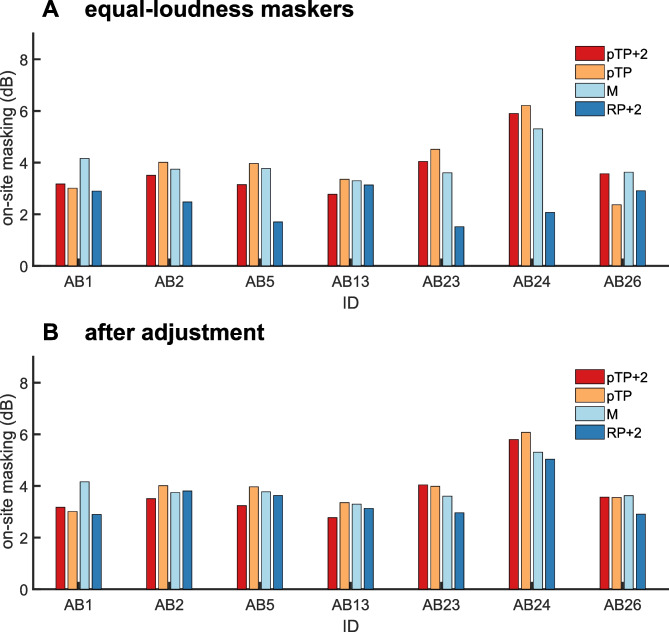
**A** Individual amount of on-site masking (same centre electrode for the probe and masker) for the different maskers, all loudness-balanced to the monopolar masker at MCL. Masking is calculated in dB between the unmasked and masked probe detection threshold. **B** Individual amount of on-site masking (same centre electrode for the probe and masker) for the different maskers, with the levels of the maskers adjusted to yield a similar within-participant amount of on-site masking across masker types

Figure 3A shows the amount of on-site masking in dB produced by equally loud maskers, obtained prior to adjusting masker levels to produce equal masking. This is of interest in light of Carlyon et al.’s [10] proposed single-point method for comparing the widths of excitation patterns produced by two maskers. They reasoned that when two maskers have equal loudness, then the one with the narrower excitation pattern should produce more on-site masking, because more of the neural excitation should be concentrated near the stimulating electrode. It can be seen that the RP + 2 masker, which we expected to produce the broadest excitation pattern based on the SCINSEVs, did indeed produce the least on-site masking. This impression was supported by the results of one-way repeated-measures ANOVA, which revealed a main effect of masker type (*F*(3,18) = 6.2, *p* = 0.03, *η*_p_^2^ = 0.51). Paired comparisons revealed that on-site masking produced by the RP + 2 masker was significantly smaller than that produced by the MP masker, after correction for multiple comparisons (*p* = 0.042). No other between-condition comparisons were significant after correction.

**Fig. 4:**
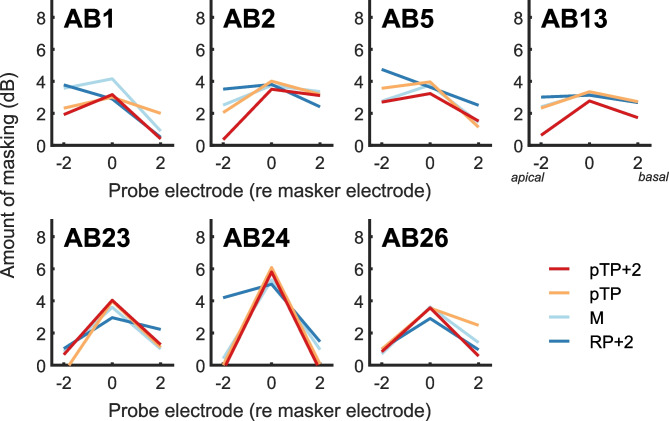
Individual excitation patterns for the various masker types (colour coding consistent across figures). Probe detection thresholds were measured with and without masker for three probe electrodes: on-site (same centre electrode as the masker) and off-site (centre electrode shifted by ± 2 electrodes); positive probe electrode numbers indicate basal measurements relative to the centre electrode of the, possibly multi-component, masker complex. The amount of masking is calculated in dB between the unmasked and masked probe detection threshold

**Fig. 5:**
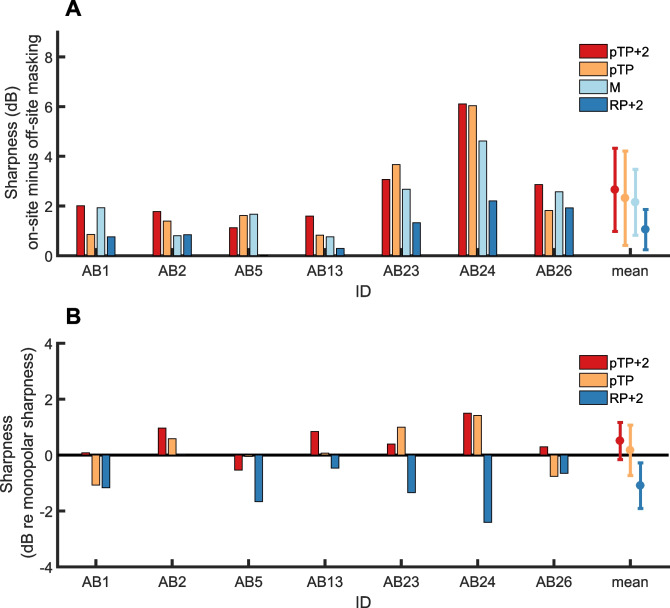
**A** Individual sharpness measure derived from the excitation patterns (average amount of masking off-site subtracted to the amount of on-site masking). Sharper excitation patterns are reflected by higher values. Error bars indicate group mean ± 1 standard deviation. **B** Individual comparisons of the sharpness measure for the different masker types with the sharpness measure in monopolar configuration. Positive values indicate sharper tuning than with monopolar, negative values indicate shallower tuning than monopolar. Error bars indicate group mean ± 1 standard deviation

Equating on-site masking was generally successful (Fig. 3B), with some exceptions including listener AB1, for whom the MP masker produced about 1 dB more masking than for all others. A 1-way repeated-measures ANOVA on the on-site masking for the four masker types found a (just) non-significant effect of masker (*F*(3,18) = 3.0, *p* = 0.06, *η*_p_^2^ = 0.33). Figure 4 shows the masked excitation patterns for the four masker types with these updated, ‘equal on-site masking’ levels. A two-way (masker type X probe electrode) repeated-measures ANOVA revealed a highly significant interaction (*F*(6,36) = 5.48; *p* < 0.001, *η*_p_^2^ = 0.48), demonstrating that the shape of the masked excitation pattern differed between masker types. There were also main effects of masker type (*F*(3,18) = 5.1, *p* = 0.01, *η*_p_^2^ = 0.46) and of probe electrode (*F*(2,12) = 9.9, *p* = 0.003, *η*_p_^2^ = 0.62). After Bonferonni correction, no pairwise comparisons between masker types were significant, but the on-site probes exhibited significantly more masking than the basal probes and marginally more masking than the apical probes (adjusted *p* values = 0.02 and 0.05, respectively). To examine this interaction further, we calculated a sharpness index for each masker, by subtracting the average of the two off-site masked thresholds from the on-site masked threshold; the results are plotted in Fig. 5A. We chose this as a simple metric that, unlike e.g. the area under (or width of) the masking pattern, does not make any assumption about the masking pattern shape. A one-way repeated-measures ANOVA revealed a significant main effect of masker type (*F*(3,18) = 7.2, *p* < 0.01, *η*_p_^2^ = 0.55), again revealing that the maskers differed in the sharpness of their excitation patterns. After Bonferroni correction for multiple comparisons, only the RP + 2 masker was found to be significantly less sharp than pTP + 2 (*p* = 0.04). Figure 5B shows the sharpness measures for each listener and for maskers RP + 2, pTP, and pTP + 2 relative to the MP masker, thereby removing effects of overall differences in selectivity between listeners.

#### Cochlear Voltage Recordings

**Fig. 6:**
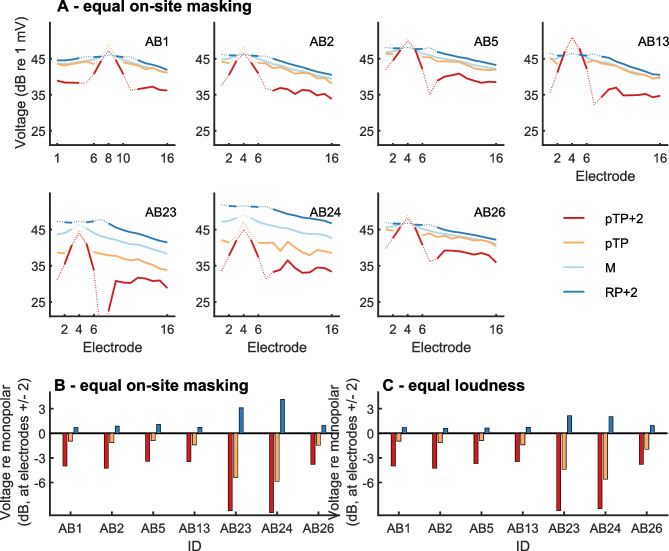
**A** Individual voltages recorded at all the non-stimulated electrodes with the various masker types (same colour code across figures) at the masker levels yielding equal on-site amount of masking. Dashed lines indicate that these are stimulated electrodes and that the values are estimated by linear extrapolation [23]. **B** Individual comparisons of the voltage at electrodes ± 2 from the centre electrode with the voltage measured in monopolar configuration. Positive values indicate broader tuning than with monopolar; negative values indicate sharper tuning than monopolar. **C** Same with the masker levels loudness-balanced to the monopolar masker at MCL

Voltage recordings for the four masker types used in the main experiment and the seven listeners are shown in Fig. 6A. The stimulus levels were set to the values obtained in the first part of the main experiment so as to produce approximately equal on-site masking by each masker. Note that data points for the central and flanking electrodes for the pTP, pTP + 2, and RP + 2 maskers are shown as thin dotted lines as these electrodes are stimulated and the recorded voltages are therefore affected by polarisation impedance and extrapolated instead [23]. Values are shown in decibels, hence ignoring any possible change in polarity, but the raw voltage values showed no flip in polarity along the electrode array. Except for points close to the centre electrode, the slope of the decay is very similar across stimulation configurations, with an overall offset that varies depending on the participant and stimulus configuration.

Figure 6B shows the average voltage in dB at positions two electrodes on either side of the stimulating electrode (same as the off-site probe centre electrodes used when measuring excitation patterns). We normalised these values to that obtained in MP configuration for each listener and with the stimulation level set so as to produce equal on-site masking for each masker type. If one assumes that the on-site voltage is similar for all masker types, then this measure can be taken as an estimate of the sharpness of the voltage profile for each stimulus type relative to that for MP. There is a consistent pattern across all participants, with off-site voltage decreasing from RP + 2, to pTP, to pTP + 2 (main effect of configuration, *F*(2, 12) = 14.9, *p* = 5e − 4, *η*_p_^2^ = 0.71). The off-site voltage was higher than produced by MP for RP + 2 and generally lower than MP for the pTP and pTP + 2 configurations. This highly consistent effect of stimulus type on voltage spread is compared to our psychophysical results with same participants in the ‘Discussion’ section. For comparison, Fig. 6C shows the same measure with the masker levels of the equal-loudness condition. Because all the voltages were measured within the limits of compliance of the implant, the difference between Fig. 6B and C is simply equal to the difference between input levels across both conditions.

Another notable feature of the SCINSEVs shown in Fig. 6A is that participants AB23 and AB24 show markedly stronger differences across stimulation configurations than the other participants. Both participants have a pre-curved electrode array (‘mid-scala’), whereas all other participants except AB26 had a straight array. However, AB26 showed a more modest effect of stimulus type on voltage spread that was similar to that of the participants implanted with a straight array. We therefore do not have enough evidence to state whether the effect of current focussing, as reflected in voltage-spread measurements, depends on array type. Some modelling studies [13] have predicted that this should be the case, but with spread of excitation from electrodes closer to the modiolus being *less* likely to be reduced by focussed stimulation, opposite to the pattern suggested by the larger between-condition differences in the data of AB23 and AB24.

## Discussion

### Comparison to Previous Studies

Overall, our results confirm that our psychophysical method is sufficiently sensitive to detect a broadening of excitation patterns in the RP + 2 configuration, but that there was no evidence for a systematic sharpening by the pTP configuration relative to the clinical-standard MP stimulus. Indeed, the only statistically significant paired comparisons from the main experiment involved condition RP + 2 (against MP for the amount of on-site masking, and against pTP + 2 for the sharpness measure), with no evidence that any of the other conditions differed systematically from each other. The lack of a substantial overall benefit for pTP stimuli in human CI listeners is consistent with the existing literature [10, 27–29], as is the finding that some individual participants (e.g. AB24, Fig. 5B) do show sharper excitation patterns for focussed (pTP, pTP + 2) compared to MP stimulation. To our knowledge, this study is the first to investigate pTP excitation patterns in humans with interleaved masking. We have argued that interleaved stimulation allows for an easier comparison with electrophysiological recordings in humans and animals than forward-masking, because one can elicit cortical onset responses without contamination by an offset response to the masker, as would occur in a forward-masking paradigm [6].

Our data are broadly consistent with the results of a study by Luo and Wu [15]. They compared pTP to pTP + 1 masking patterns, both with psychophysical forward masking and a physiological measure (electrically-evoked compound action potentials), showing a small but significant sharpening with pTP + 1. They also measured voltages at the level of the electrode array, in a similar manner to our study, namely, comparing the voltages arising from stimuli scaled to have equal loudness across conditions. This showed slightly sharper voltage patterns for pTP + 1 versus pTP near the centre of the stimulation channel, yet not as pronounced as observed here with pTP + 2. Mens and Berenstein [30] used a ‘flat TP + 2’ configuration, in which, for example, current injected via electrode 4 would be returned equally from electrodes 1, 2, 6, and 7, and reported no benefit over monopolar stimulation for speech recognition. As discussed in Guérit et al. [6], increasing the spacing between stimulating electrodes in tripolar stimulation may have the benefit of sharpening neural excitation patterns, but may also have a trade-off between how much current is returned through the side electrodes. Widening the tripolar configuration while injecting substantial amounts of current at the side electrodes might create side-lobes of excitation, in the same way as wide bipolar stimulation creates bimodal peaks of neural excitation [31, 32].

### Comparison Between Voltage Recordings and Psychophysics

Measuring the spread of voltage along the electrode array may give some insights into the neural spread of excitation. Of course, modelling studies show that neural activation actually depends on a complex interaction of factors including the distance and orientation of neurons from the stimulating electrode and on neural health, and biophysical models predict that activation is determined, to a first approximation, by the second spatial derivative of the voltage distribution along the length of the stimulated nerve [33, 34]. However a comparison of voltages at the electrode array may be informative both because they form the rationale for the use of so-called focussed forms of stimulation, such as the TP and the phased-array methods [23] and because the pattern of neural activation is unlikely to differ between two stimuli that produce similar voltages at the level of the electrode array. Importantly, the latter should be assessed by comparing the voltages at the stimulation levels used in the behavioural tasks, which we did.

If the activation of neurons near each electrode were proportional to the voltage recorded at that electrode, then the amount of off-site masking should follow the results of the voltage-spread measures. Computing a sharpness output from the voltage measures is not possible because the peak value can only be estimated and not measured. However, the voltage at electrodes ± 2 from the centre can be measured for all stimulation configurations and compared, as shown in Fig. 6B. These measures predict that if psychophysical off-site masking could be predicted from the off-site voltage, then, relative to MP, it should be greatly reduced for pTP + 2 and with a much smaller increase for RP + 2. This differs from the pattern actually observed (Fig. 5B), in which the difference between RP + 2 and MP was at least as large as that between MP and pTP + 2 (Fig. 5B). Hence, the differences between maskers in the decay of voltage from the centre masker electrode, along the electrode array, fail qualitatively to capture the differences in masked excitation patterns between these same maskers.

### Limitations and Future Directions

The excitation patterns measured here involved measurement of masked thresholds for probes presented at only 3 positions, spanning the range of 2 electrodes on either side of the masker centre electrode. It is therefore possible that further differences between masker types would have been observed if more probe positions had been tested. However, as shown in Figs. 6A and 7, the slopes of the voltage profiles were very similar for the different masker types at positions further from the masker centre electrode. As we have argued above, although a difference at the electrode level is no guarantee of a difference in the neural excitation pattern, it seems unlikely that regions of the array where the voltage profiles are very similar will produce markedly different patterns of neural activity. In addition, broadly similar results were observed with our single-point measure of masking, which, we argue, provides an estimate of spread of excitation along a wide range of the auditory nerve array [10].

The present results demonstrate the feasibility of measuring excitation patterns with an interleaved masking paradigm, and we argue that this paradigm might be used with only minor modifications to elicit cortical onset responses, permitting future comparative EEG experiments in humans and animals. Preliminary results presented by Guérit et al. [35] suggest that such measures are indeed possible, and we are currently investigating the translation of our EEG cortical-onset measures from normal-hearing cats to those implanted with a CI. It remains to be seen whether scalp recordings in cats replicate the enhanced sharpness of focussed stimulation that is evident in single-unit studies from the inferior colliculus or, instead, show the broad excitation seen in the present human psychophysical results.

## Data Availability

Data and analysis codes are available upon request.
